# Perceived behavioral control, language mindsets, and self-determination theory: a structural analysis of learning Arabic as a second language

**DOI:** 10.3389/fpsyg.2026.1861203

**Published:** 2026-08-06

**Authors:** Munassir Alhamami, Hasan Faya Alalmay

**Affiliations:** 1 English Department, College of Languages and Translation, King Khalid University, Abha, Saudi Arabia; 2 Department of Arabic Language and Literature, College of Arts and Humanities, King Khalid University, Abha, Saudi Arabia

**Keywords:** Arabic as a second language, language mindsets, multilingual language learners, perceived behavioral control, self-determination theory

## Abstract

Intensive L2 Arabic programs may place strong cognitive, emotional, and motivational demands on international students, yet limited research has examined how learners’ capability beliefs and language mindsets are related to motivation in this context. Most existing work has focused on English-dominant settings, while evidence from scholarship-based L2 Arabic programs in the Arabian Peninsula remains scarce. To address this gap, the present study tested 18 hypotheses and reports new findings from a larger funded project on learner psychology in L2 Arabic. The study examined how perceived behavioral control relates to fixed and growth language mindsets, and how these mindsets are associated with four Self-Determination Theory motivation types. A cross-sectional survey was conducted with 181 multilingual international L2 Arabic learners enrolled in an intensive program at a Saudi public university, and the data were analyzed using generalized structured component analysis. The findings showed acceptable measurement quality and supported 10 of the 18 hypotheses. Perceived behavioral control was strongly associated with higher Growth Mindsets and lower Fixed Mindsets. Fixed Mindsets were linked only to External Regulation. Growth Mindsets were strongly associated with Intrinsic Motivation and Identified Regulation and were also positively associated, though more weakly, with Introjected Regulation and External Regulation. The strongest indirect associations between perceived behavioral control and the four motivational outcomes operated through Growth Mindsets. These findings suggest that L2 Arabic teachers, program leaders, and policy makers may strengthen motivation by supporting learners’ sense of control, offering scaffolded tasks and clear feedback, and fostering growth-oriented beliefs in demanding instructional settings.

## Introduction

Learning a second language (L2) can place strong cognitive and emotional demands on learners, especially when the target language differs from their first language in script, grammar, and sociocultural norms. L2 Arabic is one such case. Learners often need to master a new alphabet, complex morphology, and diglossic variation. They also need to adapt to new academic and social settings. These demands may be stronger for scholarship students in Saudi universities because they study in intensive programs and may view their academic performance as important for future study and career plans (Alhamami, 2025a). In such settings, success may depend not only on instruction, but also on learners’ psychological resources, including perceived control, language mindsets, and motivation.

Perceived behavioral control (PBC), a central construct in the Theory of Planned Behavior (TPB), refers to learners’ judgments about their ability to perform needed behaviors and manage conditions that may affect outcomes (Ajzen, 2002, 2005). In L2 settings, PBC is closely related to self-efficacy and reflects learners’ confidence in handling tasks, overcoming difficulty, and managing study demands (Bandura, 1997). Recent work in L2 Arabic suggests that PBC is associated with intention to learn and may help explain how learners manage negative emotions, such as anxiety, in intensive programs (Alhamami, 2025b). However, less is known about how PBC is linked to broader belief systems and to different types of motivation in L2 Arabic programs.

Language mindset research offers one way to examine this link. Mindset theory distinguishes between growth beliefs, which view language ability as improvable through effort, and fixed beliefs, which view ability as largely stable (Dweck and Leggett, 1988; Lou and Noels, 2019). In language learning, mindsets may influence how learners interpret success and failure, respond to feedback, and persist after setbacks (Khajavy et al., 2021; Lou et al., 2022). Studies in English and other L2 contexts suggest that growth mindsets are related to stronger self-efficacy, engagement, and adaptive emotional patterns, while fixed mindsets are often linked to avoidance and concern with evaluation (Jiang et al., 2024; Kim, 2024; Shirvan et al., 2024). Yet much of this evidence comes from English as a foreign language (EFL) settings and from Western or East Asian contexts.

Self-Determination Theory (SDT) adds a complementary view because it focuses on motivation quality. It distinguishes autonomous motivation, including intrinsic motivation and identified regulation, from controlled motivation, including introjected regulation and external regulation (Ryan and Deci, 2017, 2020). Autonomous motivation reflects interest and personal value. Controlled motivation reflects internal pressure or external demands, such as guilt, fear of failure, grades, or rewards. Recent L2 research has used SDT to examine motivation and achievement, but it has focused mainly on English and technology-mediated learning (He and Li, 2023; Li et al., 2024).

Together, these three lines of research provide a useful model for L2 Arabic. PBC represents learners’ perceived control over language learning demands. Language mindsets represent how learners interpret ability and improvement. SDT motivation represents the quality of reasons learners report for continuing L2 study. Learners with stronger PBC may be more likely to report growth beliefs because they may view effort and strategy use as useful for improvement. Growth beliefs may also be associated with more autonomous motivation and less exclusive dependence on external pressure (Jiang et al., 2024; Lou and Noels, 2019; Zarrinabadi et al., 2022). However, these constructs may also influence each other over time. Therefore, cross-sectional evidence should be interpreted as associations rather than causal order.

Several gaps remain. First, research on language mindsets and motivation still focuses mainly on English and relatively homogeneous learner groups (Kırmızı et al., 2023; Li et al., 2025; Shirvan et al., 2024). It is not yet clear whether these findings apply to international L2 Arabic learners in Gulf universities. Second, few studies focus on scholarship students in intensive, immersion-like L2 Arabic programs with multinational cohorts. Third, prior research often tests pairwise links, rather than examining PBC, language mindsets, and SDT motivation in one model (Kim, 2024; Lou and Noels, 2017). Finally, there is limited use of structural modeling with bilingual instruments in L2 Arabic settings, despite calls for more rigorous quantitative work in this area (Alhamami, 2025b; Oruç, 2025).

The present study addresses these gaps by testing a hierarchical model that links PBC, fixed and growth language mindsets, and four SDT motivation types among international L2 Arabic learners in Saudi Arabia. The study is part of a larger project on psychological constructs in L2 Arabic programs. It focuses on male international learners enrolled in an intensive four-level program at a Saudi public university. Most learners receive full government scholarships and come from more than 30 countries. The study uses a cross-sectional survey, a bilingual Arabic-English questionnaire, and generalized structured component analysis (GSCA) to examine theory-guided direct paths and statistical indirect paths among the constructs. This design is appropriate for examining current psychological patterns in a natural educational setting. It also allows standardized data to be collected from a diverse cohort during the same instructional period, with limited disruption to teaching and learning.

This study offers three contributions. Theoretically, it links TPB and SDT through language mindsets in an underexplored L2 Arabic context. Methodologically, it examines the measurement quality of adapted bilingual scales and tests a hierarchical model with international scholarship students. Practically, it offers evidence that may help teachers and program leaders support learners’ sense of control, encourage growth-oriented beliefs, and sustain motivation in intensive programs. The next section presents the conceptual framework, research questions, and hypotheses.

### Conceptual framework, research questions, and hypotheses

The conceptual framework links learners’ capability beliefs, language mindsets, and motivational regulations in a hierarchical model. PBC was specified as an antecedent construct in the theoretical model because it reflects learners’ perceived ability to manage the academic and emotional demands of intensive L2 Arabic study. PBC was hypothesized to be associated with two higher-order constructs (HOCs), the Fixed Mindset HOC and the Growth Mindsets HOC. Each HOC reflects three lower-order constructs (LOCs): general language beliefs (GLB), second language aptitude beliefs (L2B), and age-sensitive beliefs (ASB). Stronger PBC was expected to be negatively associated with fixed beliefs and positively associated with growth beliefs.

**Figure 1:**
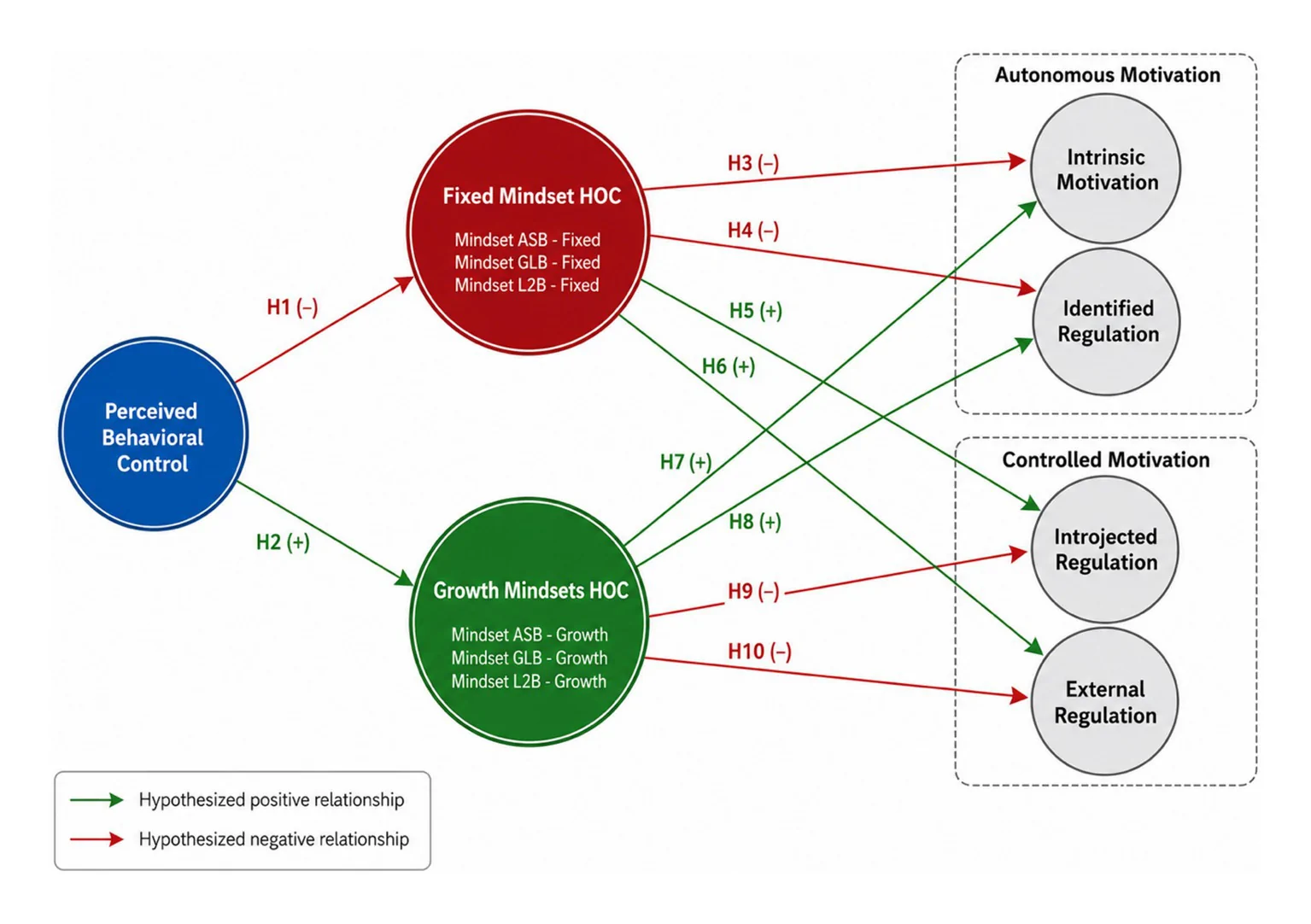
Theoretical model with direct-path hypotheses (H1-H10).

The model then specifies statistical paths from the two mindset HOCs to the four SDT motivation types. Intrinsic motivation and identified regulation represent more autonomous motivation. Introjected regulation and external regulation represent more controlled motivation (Ryan and Deci, 2017, 2020). Growth mindsets were expected to relate positively to autonomous motivation and negatively to controlled motivation. Fixed mindsets were expected to show the opposite pattern. Because the study is cross-sectional, these paths are interpreted as theory-guided associations, not as evidence of causal order. Reverse or reciprocal relations are also possible. For example, Growth Mindsets may also strengthen learners’ sense of control over time. Figure 1 presents the theoretical model.

Based on this framework, the study addresses the following research questions:

*RQ1:* How is PBC related to the Fixed Mindset HOC and the Growth Mindsets HOC among international L2 Arabic learners?

*RQ2:* How are the Fixed Mindset HOC and the Growth Mindsets HOC related to the four SDT motivation types among international L2 Arabic learners?

*RQ3:* Do the Fixed Mindset HOC and the Growth Mindsets HOC show statistical indirect paths between PBC and the four SDT motivation types among international L2 Arabic learners?

To answer these questions, the model specifies 18 hypotheses. The first ten hypotheses concern direct paths among the constructs, as shown in Figure 1. The remaining eight hypotheses concern statistical indirect paths from PBC to the four motivation types through the two mindset HOCs.

### Paths from PBC

*H1:* PBC is negatively associated with the Fixed Mindset HOC.

*H2:* PBC is positively associated with the Growth Mindsets HOC.

### Paths from the fixed mindset HOC to SDT motivation

*H3:* The Fixed Mindset HOC is negatively associated with intrinsic motivation.

*H4:* The Fixed Mindset HOC is negatively associated with identified regulation.

*H5:* The Fixed Mindset HOC is positively associated with introjected regulation.

*H6:* The Fixed Mindset HOC is positively associated with external regulation.

### Paths from the growth mindsets HOC to SDT motivation

*H7:* The Growth Mindsets HOC is positively associated with intrinsic motivation.

*H8:* The Growth Mindsets HOC is positively associated with identified regulation.

*H9:* The Growth Mindsets HOC is negatively associated with introjected regulation.

*H10:* The Growth Mindsets HOC is negatively associated with external regulation.

### Eight further hypotheses specify statistical indirect paths from PBC to the four SDT motivation types through the two mindset HOCs

*H11:* The Fixed Mindset HOC shows a statistical indirect path between PBC and intrinsic motivation.

*H12:* The Growth Mindsets HOC shows a statistical indirect path between PBC and intrinsic motivation.

*H13:* The Fixed Mindset HOC shows a statistical indirect path between PBC and identified regulation.

*H14:* The Growth Mindsets HOC shows a statistical indirect path between PBC and identified regulation.

*H15:* The Fixed Mindset HOC shows a statistical indirect path between PBC and introjected regulation.

*H16:* The Growth Mindsets HOC shows a statistical indirect path between PBC and introjected regulation.

*H17:* The Fixed Mindset HOC shows a statistical indirect path between PBC and external regulation.

*H18:* The Growth Mindsets HOC shows a statistical indirect path between PBC and external regulation.

### Literature review

#### PBC and language mindsets (H1-H2)

PBC is a central construct in TPB. It refers to learners’ judgments about their ability to perform a behavior and their control over conditions that may support or constrain it (Ajzen, 2002, 2005). In language education, PBC is closely related to academic self-efficacy because it reflects learners’ beliefs that they can manage tasks, overcome difficulties, and meet the demands of L2 learning (Bandura, 1997; Chai et al., 2023; Huang and Liu, 2026). In L2 Arabic, previous work suggests that PBC is associated with intention to learn and may help explain how learners manage anxiety and frustration in intensive programs (Alhamami, 2019, 2025b).

Language mindset theory offers a related view of capability beliefs. It distinguishes between growth beliefs, which view language ability as improvable, and fixed beliefs, which view ability as largely stable (Dweck and Leggett, 1988; Dweck and Molden, 2017). In L2 learning, mindsets are linked to how learners interpret success, failure, feedback, and difficulty (Lou and Noels, 2016, 2017, 2019; Oruç, 2025). Growth mindsets are often associated with mastery goals, challenge seeking, adaptive emotion regulation, and engagement (Jiang et al., 2024; Lou et al., 2022; Sadoughi et al., 2023). Fixed mindsets are more often associated with avoidance, helpless responses, and concern with self-worth (Khajavy et al., 2021; Kırmızı et al., 2023; Shirvan et al., 2024).

Several studies connect these two areas. Evidence across L2 contexts suggests that self-efficacy and PBC are positively associated with growth mindsets and negatively associated with fixed mindsets (Derakhshan and Fathi, 2024; Ho et al., 2025; Kim, 2024; Zhai and Li, 2025; Zhang et al., 2024; Zarrinabadi et al., 2022, 2024). Learners who believe that effort can lead to progress may also report stronger perceived control over learning. By contrast, learners with weaker PBC may be more likely to report fixed beliefs, especially in demanding contexts. Based on this evidence, PBC is expected to be negatively associated with the Fixed Mindset HOC (H1) and positively associated with the Growth Mindsets HOC (H2).

#### Fixed mindsets and SDT motivation (H3-H6)

SDT distinguishes motivation types from more autonomous to more controlled regulation (Ryan and Deci, 2017, 2020). Intrinsic motivation and identified regulation reflect more autonomous reasons for learning. Introjected regulation and external regulation reflect more controlled reasons. In L2 research, autonomous motivation is often linked to persistence, achievement, and well-being, while controlled motivation is often linked to more fragile engagement and higher stress (He and Li, 2023; Li et al., 2024; Uhing et al., 2025).

Fixed language mindsets may be relevant to this motivation continuum. When learners view ability as difficult to change, they may interpret failure as evidence of limited ability rather than as feedback for improvement (Dweck and Leggett, 1988; Lou and Noels, 2019). This interpretation may weaken competence beliefs and reduce interest or personal value in learning (Ryan and Deci, 2017). Studies in several L2 settings suggest that fixed mindsets are negatively related to intrinsic motivation and enjoyment, and are also linked to lower engagement and higher anxiety (Jiang et al., 2024; Kırmızı et al., 2023; Li et al., 2025; Shirvan et al., 2024). Therefore, the Fixed Mindset HOC is expected to be negatively associated with intrinsic motivation (H3) and identified regulation (H4).

Fixed mindsets may also relate to controlled motivation. Learners who hold fixed beliefs may focus more on evaluation, grades, and avoiding negative judgments (Lou and Noels, 2016, 2019; Lou et al., 2017). These tendencies fit introjected regulation, where effort is linked to guilt or shame, and external regulation, where effort is linked to rewards, grades, or expectations (Kashefian-Naeeini et al., 2024; Li et al., 2024; Uhing et al., 2025). Prior L2 studies also suggest links between fixed beliefs and obligation-focused motives (Kırmızı et al., 2023; Lou and Noels, 2017, 2019). Thus, the Fixed Mindset HOC is expected to be positively associated with introjected regulation (H5) and external regulation (H6).

#### Growth mindsets and SDT motivation (H7-H10)

Growth mindsets assume that language ability can improve through effort, strategies, and supportive feedback (Dweck and Molden, 2017; Lou and Noels, 2019). This belief system may support competence, challenge seeking, and resilience during difficulty (Jiang et al., 2024; Zarrinabadi et al., 2022; Zhang et al., 2024). In L2 research, growth mindsets have been associated with enjoyment, engagement, and willingness to communicate (Derakhshan and Fathi, 2024; Eerdemutu et al., 2024; Fan and Wang, 2025; Ho et al., 2025). These outcomes fit the more autonomous side of SDT.

Several studies suggest that growth mindsets are positively related to intrinsic motivation and identified regulation. Learners who believe they can improve may find language learning more interesting and may view it as useful for their long-term goals (Jiang et al., 2024; Li et al., 2024; Sadoughi et al., 2023). Therefore, the Growth Mindsets HOC is expected to be positively associated with intrinsic motivation (H7) and identified regulation (H8).

Growth beliefs may also be linked to weaker reliance on controlled motives. When learners treat mistakes as part of learning, they may depend less on guilt, shame, or fear of judgment to sustain effort (Lou and Noels, 2016, 2019). Research outside L2 contexts has reported negative relations between growth mindsets and maladaptive internal pressure (Burnette et al., 2013; Renaud-Dubé et al., 2015, as cited in Fan and Wang, 2025). L2 studies also link growth mindsets with more adaptive emotional profiles and lower anxiety (Hu et al., 2025; Zarrinabadi et al., 2024). Based on this logic, the Growth Mindsets HOC is expected to be negatively associated with introjected regulation (H9) and external regulation (H10).

#### Mindsets as statistical mediators between PBC and motivation (H11-H18)

Language mindsets can be understood as meaning systems related to goals, feedback, and motivation patterns (Lou and Noels, 2017, 2019; Oruç, 2025). SDT also emphasizes that motivation is linked to learners’ interpretations of competence, autonomy, and relatedness (Ryan and Deci, 2017, 2020). These ideas suggest that PBC may be statistically related to motivation through mindset systems that reflect how learners understand learning difficulty and improvement.

Several studies support this view. Growth mindsets have been reported as indirect links between autonomy-supportive teaching and engagement, adaptability, and positive emotions (Jiang et al., 2024; Sadoughi et al., 2023; Zarrinabadi et al., 2022). Other research suggests that malleability beliefs may connect broader self-beliefs and contextual factors with motivational outcomes (Fan and Wang, 2025; Li et al., 2025). Meta-analytic evidence outside language education also indicates that mindsets often function as intermediate variables between competence beliefs, goals, and self-regulation (Burnette et al., 2013).

Building on this work, the present model treats PBC and language mindsets as related layers of learner psychology. Higher PBC may be associated with stronger growth beliefs and weaker fixed beliefs. These mindset systems may then be linked to whether learners report more internalized reasons for learning or stronger reliance on external pressure. The model therefore tests whether the Fixed Mindset HOC and the Growth Mindsets HOC show statistical indirect paths between PBC and the four SDT motivation types (H11-H18).

#### L2 Arabic context

Although PBC, language mindsets, and SDT motivation are well established in psychology and L2 research, they remain underexplored in L2 Arabic programs. Existing Arabic-related research has examined motivation, self-efficacy, and affect, but it often focuses on single variables or specific interventions (Calafato, 2023; Eltahir et al., 2021; Hammad, 2019; Ramezanzadeh et al., 2025). Other studies have examined international students’ motivation, peace of mind, and perceived proficiency in Saudi universities (Alenezi, 2025; Venugopal Muthuswamy, 2023). However, few studies bring PBC, fixed and growth mindsets, and multiple SDT motivation types into one model.

This study addresses this gap with international scholarship students in an intensive L2 Arabic program in Saudi Arabia. It uses bilingual Arabic-English measures and GSCA to test a hierarchical model with 18 hypotheses. The cross-sectional survey design is suitable for this purpose because the study examines current theory-guided associations in a natural educational setting, not causal change over time. In doing so, the study extends work on mindsets and motivation beyond EFL-dominant settings and offers evidence that may inform intensive L2 Arabic teaching and program design.

### Methodology

#### Context

This study was conducted in a Department of Arabic Language Teaching for Non-Native Speakers at a public university in southern Saudi Arabia. Students were admitted through the Study in Saudi platform, a national online portal launched in 2022 to manage applications and scholarship procedures for international students. The department offers a two-year intensive L2 Arabic diploma with four sequential levels, one level per semester. Courses are taught in Arabic by specialist instructors from several Arab countries, with Saudi faculty forming the majority. Most students receive scholarships that cover tuition, housing, monthly allowances, and annual travel tickets. Many plan to continue into degree programs in Saudi universities after completing the diploma. New students complete written and oral placement tests on arrival and progress by passing end-of-level examinations. At the time of data collection, the department admitted only male students, so all participants were men. This article is part of a larger project on psychological constructs in L2 Arabic programs.

#### Study design

The study used a cross-sectional quantitative survey design. This design was selected because the study aimed to examine theory-guided associations among PBC, language mindsets, and SDT motivation in a natural intensive L2 Arabic program. The purpose was not to test an intervention or trace individual change over time. Rather, the study aimed to capture learners’ current beliefs, perceptions, and motivation during the same instructional period.

This design was appropriate for the study purpose. Cross-sectional survey designs are widely used in education, educational psychology, and L2 research when researchers examine psychological and educational constructs such as motivation, beliefs, anxiety, self-efficacy, attitudes, perceived competence, and burnout (Dörnyei and Taguchi, 2010; Mackey and Gass, 2015). They are also useful when the target constructs are not directly observable and need to be measured through multi-item scales. In this study, the design allowed the researchers to collect standardized data from learners across the four program levels with limited disruption to teaching and learning. Therefore, the findings are interpreted as current patterns and theory-guided associations, not as evidence of causal order or developmental change.

#### Participants

After screening, the final sample included 181 international L2 Arabic learners enrolled in the four-level intensive diploma program. All participants were male, reflecting the department’s admission policy at the time. Students were distributed across levels as follows: Level 1 (*n* = 21, 11.6%), Level 2 (*n* = 42, 23.2%), Level 3 (*n* = 64, 35.4%), and Level 4 (*n* = 54, 29.8%). Learners reported more than 30 national backgrounds. The largest groups were Ivorian (*n* = 33, 18.2%) and Nigerian (*n* = 26, 14.4%), with smaller groups spread across Africa, Asia, and Europe. Most participants were scholarship students funded by the Saudi Ministry of Education. Most planned to major in Islamic Studies (*n* = 116, 64.1%), followed by Arabic Language Studies (*n* = 24, 13.3%) and Computer Sciences (*n* = 13, 7.2%). Most intended to complete a bachelor’s degree (*n* = 147, 81.2%), while 10 (5.5%) intended a master’s degree and 24 (13.3%) intended a doctorate.

#### Instruments

Data came from a longer bilingual questionnaire that covered several psychological constructs in L2 Arabic learning. This article focused on PBC, SDT motivation, and language mindsets. PBC was measured with four items adapted from a validated L2 Arabic scale (Alhamami, 2025b). SDT motivation was measured with 20 items adapted from Takahashi and Im (2020), with five items each for intrinsic motivation, identified regulation, introjected regulation, and external regulation. Language mindsets were measured with 18 items from the Language Mindsets Inventory (Lou and Noels, 2019), adapted to L2 Arabic. The items covered GLB, L2B, and ASB, with fixed and growth items in each domain. In the model, fixed items were represented under the Fixed Mindset HOC, and growth items were represented under the Growth Mindsets HOC.

All items used a six-point Likert scale, from 1 = strongly agree to 6 = strongly disagree, with no neutral midpoint. The same response format was used across constructs to support consistency. The even-number scale was used to reduce midpoint responding and encourage clearer choices. Because lower raw scores indicated stronger agreement, item direction was considered when interpreting the descriptive results and the structural paths.

#### Instrument adaptation procedures

All instruments were adapted from established measures with minor wording changes to fit the L2 Arabic context while preserving the original construct meanings. The first author adapted the English items by replacing references to English or foreign language learning with L2 Arabic and the local program. He then prepared parallel Arabic and English versions for each item. Three master’s students in applied linguistics reviewed the bilingual items for clarity, equivalence, and cultural appropriateness. Based on their feedback, minor wording changes were made.

The items were then uploaded into a Google Form. The second author reviewed the full online survey for content, face validity, flow, and layout. Five L2 Arabic students also piloted the online form and reported unclear wording or technical issues. Their feedback led to small refinements in phrasing and formatting without changing the construct meanings. These procedures supported content and face validity before the main analysis.

#### Data collection and ethics

The protocol was approved by the university Research Ethics Committee before data collection (approval number ECM#2025–3,101). All enrolled students in the L2 Arabic program were invited to participate. Data collection took place during the student activity period to avoid disrupting classes. Students completed the survey by level across Weeks 10 and 11 of the semester, with each level assigned a separate session in a large campus hall. A questionnaire was suitable for this study because it allowed comparable data to be collected from students across all levels at the same stage of the semester. Questionnaires are widely used in second language research because they can collect a large amount of information in a short time and produce data that can be processed systematically (Brown, 2001; Dörnyei and Taguchi, 2010).

The participants were informed that participation was voluntary, that non-participation had no academic consequences, and that they could withdraw at any time without giving a reason. Responses were anonymous, stored securely, and used only for research. Students accessed the survey through a link and a QR code and completed it on their own devices. Computer labs were available for students without suitable devices. The bilingual format allowed students to read items in Arabic, English, or both. Each session lasted about one hour. Students could take short breaks because the full questionnaire included constructs beyond those analyzed in this article. Light refreshments were provided by the university administration, and no financial incentives were offered.

#### Data cleaning and analysis

The initial dataset included 202 completed surveys. To screen for unengaged responding, the response range for each participant was calculated on the 18 mindset items. A range of zero indicated that the same option had been selected for all items, and 15 cases were removed. The same screening was then applied to the 20 SDT motivation items, and six additional cases were removed. The final sample included 181 participants. The Google Form required responses to all items, so there were no missing data.

The model was estimated using the GSCA algorithm in SmartPLS 4 (Ringle et al., 2024). SmartPLS 4 was used as the software platform, but the analysis was not conducted with the standard partial least squares structural equation modeling (PLS-SEM) algorithm. In this study, GSCA refers to the specific estimator, while component-based structural equation modeling (SEM) refers to the broader methodological family to which GSCA belongs. Each mindset HOC was modeled using the relevant fixed or growth mindset indicators from the three domains, GLB, L2B, and ASB.

The analysis was reported in two stages. First, the measurement model was evaluated using outer loadings, internal consistency reliability, composite reliability, average variance extracted (AVE), and discriminant validity. Common reference values were used, including Cronbach’s alpha and composite reliability values of at least 0.70 and AVE values of at least 0.50 (Hair et al., 2022; Kline, 2016). Discriminant validity was assessed using the upper bounds of the two-tailed 95% bootstrap confidence intervals of latent correlations, following Rönkkö and Cho (2022). Second, the structural model was evaluated using path coefficients, explained variance, and inner variance inflation factor (VIF) values. Bootstrapping with 10,000 subsamples was used to estimate standard errors and test the significance of direct and indirect paths.

A separate factor-based confirmatory factor analysis (CFA) was not treated as a required preliminary stage because CFA and GSCA represent constructs differently. CFA treats constructs as common factors and examines how well a factor model reproduces the observed covariance matrix. In contrast, GSCA defines constructs as weighted components and estimates the measurement, structural, and weighted relation models within one unified framework (Hwang and Takane, 2014). A CFA would therefore test a different measurement model rather than provide an additional stage of the component-based GSCA analysis. The adaptation involved contextual and bilingual wording while retaining the intended construct meanings. Evidence for measurement quality in the L2 Arabic sample was obtained through bilingual review, expert and student feedback, pilot testing, and the GSCA measurement model. The analysis examined outer loadings and weights, reliability, average variance extracted, bootstrap-based discriminant validity, and collinearity. A CFA using the same 181 participants would provide a supplementary same-sample analysis, not independent validation. However, factor-based validation with a new sample would provide useful additional evidence. This limitation is acknowledged below.

#### Analytical approach and rationale for GSCA

GSCA was selected instead of standard PLS-SEM or covariance-based structural equation modeling (CB-SEM). This choice was methodological and theoretical, not only based on sample size. GSCA is a component-based SEM approach that estimates associations among weighted components created from observed indicators (Hwang and Takane, 2014). This approach differs from CB-SEM, where constructs are usually represented as common factors and the main aim is to reproduce the observed covariance matrix. It also differs from standard PLS-SEM, although both GSCA and PLS-SEM belong to the wider component-based SEM family.

GSCA was appropriate because the study examined theory-informed associations among operational scale composites in an integrated hierarchical model. The model included PBC, two mindset HOCs, and four SDT motivation outcomes, with several direct and indirect paths. The constructs were measured with established questionnaire scales adapted to the L2 Arabic context and represented operationally as weighted components. GSCA therefore matched the construct representation and the purpose of the analysis better than CB-SEM, which would be more suitable if the main aim were to test a common-factor model. GSCA also allowed the study to assess measurement quality, structural paths, indirect paths, and GSCA-relevant model fit information within one framework. For this reason, the study reports GSCA fit indices, including SRMR, FIT, AFIT, FITs, and FITm, rather than CB-SEM indices such as χ^2^, CFI, TLI, and RMSEA.

#### Assessment of common method variance

Because the data were collected through self-report measures in one survey session, common method variance was considered as a possible source of bias. Several procedural steps were used to reduce this risk. Participation was voluntary, responses were anonymous, and students were informed that non-participation had no academic consequences. The bilingual format allowed students to read the items in Arabic, English, or both, which helped reduce possible misunderstanding. The questionnaire was also reviewed, piloted, and refined before data collection. During data cleaning, unengaged response patterns were screened and removed.

As a statistical diagnostic, Harman’s single-factor test was conducted using the 42 retained indicators included in the reported model. The first unrotated factor explained 43.65% of the total variance, which was below the common 50% reference point. This result suggests that a single method factor did not dominate the data. However, this test was treated as a preliminary diagnostic rather than definitive evidence because common method variance cannot be fully ruled out in cross-sectional self-report research.

## Results

### Descriptive statistics and interpretation of mean scores

All constructs were measured on a six-point scale, from 1 = strongly agree to 6 = strongly disagree. Higher scores therefore indicated stronger disagreement, and lower composite means indicated stronger agreement with the item statements. To interpret the composite means, the equal-interval method was used. The interval width was calculated as (6–1) / 6 = 0.83. Mean scores were classified as 1.00–1.83 (very high), 1.84–2.67 (high), 2.68–3.51 (moderate-high), 3.52–4.33 (moderate-low), 4.34–5.17 (low), and 5.18–6.00 (very low). Table 1 reports the descriptive statistics and equal-interval classifications for all constructs and mindset domains.

**Table 1:** Equal-interval interpretation of the study constructs (*n* = 181).

Construct	Mean	SD	Descriptive level
External regulation	3.45	1.42	Moderate-high
Identified regulation	2.12	1.57	High
Intrinsic motivation	2.24	1.54	High
Introjected regulation	2.70	1.43	Moderate-high
PBC	2.17	1.55	High
Fixed mindset HOC	3.69	1.36	Moderate-low
Growth mindsets HOC	2.10	1.34	High
Mindset ASB, fixed	3.70	1.54	Moderate-low
Mindset ASB, growth	2.15	1.41	High
Mindset GLB, fixed	3.60	1.51	Moderate-low
Mindset GLB, growth	2.11	1.40	High
Mindset L2B, fixed	3.76	1.58	Moderate-low
Mindset L2B, growth	2.04	1.37	High

Lower means indicate stronger agreement with the construct statements. Descriptive levels were interpreted according to agreement with construct content.

### Measurement model

The final analytic sample included 181 learners. All measurement and structural results were obtained from the GSCA algorithm in SmartPLS 4. Outer loadings were inspected first. PBC items showed very strong loadings (0.91–0.96). For the SDT motivation constructs, loadings ranged from 0.59 to 0.95. Identified Regulation and Intrinsic Motivation showed very strong indicators, with most loadings above 0.90. Introjected Regulation and External Regulation showed acceptable loadings that were generally above 0.70. One External Regulation item loaded at 0.59. It was retained because it represented a theoretically important aspect of external pressure, and the construct met reliability and AVE criteria. For language mindsets, Fixed Mindset HOC items loaded from 0.69 to 0.78, while Growth Mindsets HOC items loaded from 0.85 to 0.89. Overall, the loading pattern supported adequate measurement quality.

Outer weights were also examined to check each indicator’s contribution. The weights were positive and broadly similar across indicators, ranging from about 0.17 to 0.36. For PBC, items reflecting general capability showed slightly larger weights than more specific statements. For SDT motivation, items that closely reflected each regulation type tended to carry slightly larger weights, while other items still contributed meaningfully. For mindsets, items across GLB, L2B, and ASB showed balanced weights. This suggests that no single mindset domain dominated the HOCs. Taken together, the loading and weight results supported retaining all items.

### Reliability and convergent validity

Reliability and convergent validity were then assessed. As shown in Table 2, Cronbach’s alpha ranged from 0.79 to 0.96, composite reliability ranged from 0.86 to 0.97, and AVE ranged from 0.53 to 0.89. These values met common reference criteria and indicated adequate to strong reliability and convergent validity.

**Table 2:** Reliability and convergent validity.

Construct	Cronbach’s alpha	Composite reliability (rho_c)	AVE
External regulation	0.79	0.86	0.55
Fixed mindset HOC	0.89	0.91	0.53
Growth mindsets HOC	0.96	0.97	0.76
Identified regulation	0.95	0.96	0.84
Intrinsic motivation	0.92	0.94	0.76
Introjected regulation	0.84	0.88	0.61
PBC	0.96	0.97	0.89

The reliability values were also compared with the source studies. Alhamami (2025b) reported strong reliability for PBC, with *α* = 0.896, CR = 0.928, and AVE = 0.763. Takahashi and Im (2020) reported *α* = 0.94 for Intrinsic Motivation, *α* = 0.88 for Identified Regulation, *α* = 0.82 for Introjected Regulation after item deletion, and *α* = 0.71 for External Regulation after item deletion. The supplementary materials accompanying Lou and Noels (2019), based on Lou and Noels (2017), reported *α* = 0.82 for Fixed language mindsets, *α* = 0.90 for Growth language mindsets, and *α* = 0.91 for the composite score. In the present study, the adapted L2 Arabic measures showed comparable or stronger reliability, with *α* values ranging from 0.79 to 0.96 and CR values ranging from 0.86 to 0.97.

### Discriminant validity

Discriminant validity was assessed following Rönkkö and Cho (2022). The analysis used the upper bounds of the two-tailed 95% bootstrap confidence intervals of the latent correlations obtained from the GSCA model, with 10,000 bootstrap resamples. Under this approach, upper confidence limits below 0.80 indicate no problem, values between 0.80 and 0.90 indicate a marginal problem, values between 0.90 and 1.00 indicate a moderate problem, and correlations that are not empirically distinguishable from 1.00 indicate a severe problem.

The results showed no severe discriminant validity problem for any construct pair. Most pairs were in the no-problem range, such as Fixed Mindset HOC with External Regulation, 95% CI [0.23, 0.53], Identified Regulation with External Regulation, 95% CI [0.08, 0.40], Intrinsic Motivation with External Regulation, 95% CI [0.05, 0.37], and PBC with Introjected Regulation, 95% CI [0.49, 0.73]. Several theoretically related pairs fell in the marginal range, including Identified Regulation with Growth Mindsets HOC, 95% CI [0.70, 0.88], Intrinsic Motivation with Growth Mindsets HOC, 95% CI [0.73, 0.88], PBC with Growth Mindsets HOC, 95% CI [0.71, 0.89], PBC with Identified Regulation, 95% CI [0.65, 0.87], and PBC with Intrinsic Motivation, 95% CI [0.69, 0.89]. The strongest association was between Intrinsic Motivation and Identified Regulation, *r* = 0.93, 95% CI [0.89, 0.95]. This result indicates substantial empirical overlap between two adjacent autonomous motivation constructs. Although the confidence interval remained below 1.00, the empirical separation between these two constructs was limited in the present sample. Accordingly, results involving these two constructs should not be treated as fully independent evidence. This overlap may reflect the scholarship-based L2 Arabic context, where interest in learning Arabic and personal value for learning Arabic may be closely connected. Therefore, findings involving Intrinsic Motivation and Identified Regulation should be interpreted with particular caution. In this study, these variables may be best understood as closely related forms of autonomous motivation rather than fully independent motivational constructs.

Collinearity was also examined in the inner model. VIF values ranged from 1.00 to 1.05, which suggests that multicollinearity was not a concern.

### Model fit

GSCA model fit was evaluated using the fit indices produced by the GSCA algorithm in SmartPLS 4. As shown in Table 3, SRMR was 0.09 for the saturated model and 0.12 for the estimated model. FIT and AFIT values were 0.65 and 0.64, respectively. FITs and FITm were 0.37 and 0.69. These values provide mixed but usable evidence for model evaluation. The estimated SRMR of 0.12 is above the commonly suggested GSCA reference value of 0.08 for samples larger than 100 (Cho et al., 2020). This suggests that some residual misfit may remain and that the model should not be described as having strong global fit.

**Table 3:** GSCA model fit indices.

Global fit index	Saturated model	Estimated model
SRMR	0.09	0.12
FIT	0.00	0.65
AFIT	0.00	0.64
FITs	0.00	0.37
FITm	0.00	0.69

SRMR, standardized root mean square residual; FIT, overall fit index; AFIT,adjusted fit index; FITs, structural model fit index; FITm, measurement model fit index. Fit indices were obtained from the GSCA output in SmartPLS 4. The estimated SRMR was above the commonly suggested GSCA reference value and was therefore interpreted cautiously. FIT, AFIT, FITs, and FITm were used as complementary GSCA fit information rather than as fixed pass or fail criteria. CB-SEM fit indices were not produced because the model was estimated using GSCA.

At the same time, SRMR should not be used as a single rejection rule. GSCA fit indices assess different aspects of model quality. FIT and AFIT indicate the proportion of variance explained by the model, whereas SRMR reflects residual differences between observed and model-implied covariances. In the present study, FIT = 0.65 and AFIT = 0.64 suggest that the model explained a meaningful proportion of total variance. FITm = 0.69 suggests meaningful explanatory quality in the measurement part of the model. FITs = 0.37 suggests more moderate explanatory quality in the structural part and supports cautious interpretation of the structural paths. Taken together, the model fit evidence should be interpreted as mixed but usable, especially when considered with the reliability results, validity evidence, theoretical basis, and substantive pattern of findings.

Because the model was estimated using GSCA in SmartPLS 4, the study reports GSCA-relevant indices, including SRMR, FIT, AFIT, FITs, and FITm. Common CB-SEM fit indices, such as χ^2^, CFI, TLI, and RMSEA, were not reported because they are linked mainly to common-factor CB-SEM estimation and were not produced by the GSCA algorithm. Reporting them would require estimating a different model with different construct assumptions.

### Structural model

Because the measurement model met the main criteria for reliability, convergent validity, discriminant validity, and collinearity, the structural model was then examined. Bootstrapping with 10,000 resamples was used to estimate standard errors and test the significance of direct and indirect paths. The interpretation of path coefficients was based on agreement with item content, because lower raw scores indicated stronger endorsement of the construct.

**Figure 2:**
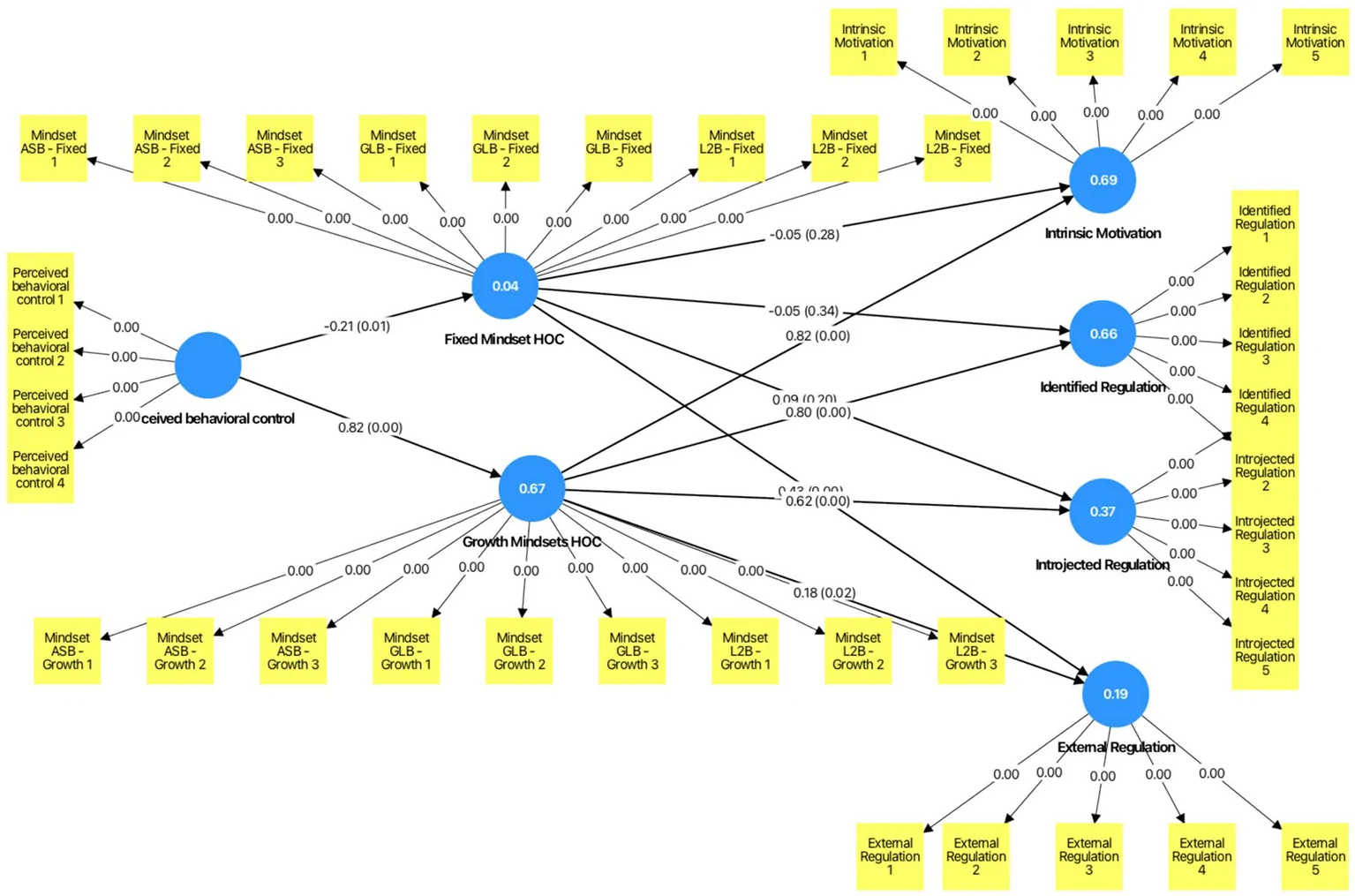
Structural model results.

Figure 2 and Table 4 show the direct path results. PBC showed a strong positive path to Growth Mindsets HOC and a weaker negative path to Fixed Mindset HOC. PBC was strongly associated with Growth Mindsets HOC (*β* = 0.82, *p* < 0.001) and weakly associated with lower Fixed Mindset HOC (*β* = −0.21, *p* = 0.01). These paths explained a small share of variance in Fixed Mindset HOC (R^2^ = 0.04) and a large share of variance in Growth Mindsets HOC (R^2^ = 0.67).

**Table 4:** Direct path coefficients.

Path	*β*	*M*	SD	*t*	*p*
Fixed Mindset HOC → External regulation	0.43	0.44	0.07	6.19	< 0.001
Fixed mindset HOC → Identified regulation	−0.05	−0.05	0.05	0.96	0.34
Fixed mindset HOC → Intrinsic motivation	−0.05	−0.05	0.05	1.07	0.28
Fixed mindset HOC → Introjected regulation	0.09	0.10	0.07	1.27	0.20
Growth mindsets HOC → External regulation	0.18	0.19	0.08	2.28	0.02
Growth mindsets HOC → Identified regulation	0.80	0.81	0.05	15.33	< 0.001
Growth mindsets HOC → Intrinsic motivation	0.82	0.82	0.04	18.31	< 0.001
Growth mindsets HOC → Introjected regulation	0.62	0.63	0.06	10.70	< 0.001
PBC → Fixed mindset HOC	−0.21	−0.21	0.08	2.70	0.01
PBC → Growth mindsets HOC	0.82	0.82	0.04	18.76	< 0.001

The two mindset HOCs showed different associations with the four SDT motivation types. Fixed Mindset HOC showed a significant positive path only to External Regulation (*β* = 0.43, *p* < 0.001). Its paths to Intrinsic Motivation, Identified Regulation, and Introjected Regulation were small and not significant (βs = −0.05 to 0.09, ps > 0.05). In contrast, Growth Mindsets HOC was strongly and positively associated with Intrinsic Motivation (*β* = 0.82, *p* < 0.001) and Identified Regulation (*β* = 0.80, *p* < 0.001). It also showed a moderate positive path to Introjected Regulation (*β* = 0.62, *p* < 0.001) and a smaller positive path to External Regulation (*β* = 0.18, *p* = 0.02). The model explained substantial variance in Intrinsic Motivation (R^2^ = 0.69) and Identified Regulation (R^2^ = 0.66), moderate variance in Introjected Regulation (R^2^ = 0.37), and a smaller share in External Regulation (R^2^ = 0.19).

### Specific indirect paths

Specific indirect paths are reported in Table 5. The strongest indirect associations between PBC and the four motivation outcomes operated through Growth Mindsets HOC. The indirect paths through Growth Mindsets HOC were significant for External Regulation (*β* = 0.15, *p* = 0.03), Identified Regulation (*β* = 0.66, *p* < 0.001), Intrinsic Motivation (*β* = 0.67, *p* < 0.001), and Introjected Regulation (*β* = 0.51, *p* < 0.001). The indirect paths through Fixed Mindset HOC were small and not significant for Intrinsic Motivation, Identified Regulation, and Introjected Regulation (ps > 0.05). The only significant indirect path through Fixed Mindset HOC involved External Regulation (*β* = −0.09, *p* = 0.02). Overall, PBC was statistically related to the motivation outcomes mainly through Growth Mindsets HOC, while Fixed Mindset HOC showed a narrower indirect association with External Regulation.

**Table 5:** Specific indirect paths.

Path	*β*	*M*	SD	*t*	*p*
PBC → Growth Mindsets HOC → External Regulation	0.15	0.16	0.07	2.24	0.03
PBC → Fixed Mindset HOC → External Regulation	−0.09	−0.09	0.04	2.40	0.02
PBC → Growth Mindsets HOC → Identified Regulation	0.66	0.67	0.07	10.07	< 0.001
PBC → Fixed Mindset HOC → Identified Regulation	0.01	0.01	0.01	0.79	0.43
PBC → Growth Mindsets HOC → Intrinsic Motivation	0.67	0.68	0.06	10.89	< 0.001
PBC → Fixed Mindset HOC → Intrinsic Motivation	0.01	0.01	0.01	0.88	0.38
PBC → Growth Mindsets HOC → Introjected Regulation	0.51	0.52	0.06	8.27	< 0.001
PBC → Fixed Mindset HOC → Introjected Regulation	−0.02	−0.02	0.02	1.11	0.27

## Discussion

This study examined how international L2 Arabic learners’ PBC, language mindsets, and SDT motivation were related within one structural model. Using adapted bilingual instruments and GSCA in SmartPLS 4, the study tested 18 hypotheses in an intensive scholarship-based L2 Arabic program at a Saudi public university.

The descriptive results suggest that many learners reported strong growth-oriented beliefs across the mindset domains and weaker endorsement of fixed beliefs. This pattern is consistent with mindset theory, which links growth beliefs with persistence and more adaptive responses to difficulty (Dweck and Leggett, 1988; Lou and Noels, 2019). Learners also reported relatively strong PBC and more self-determined reasons for learning. This aligns with TPB and SDT, which stress the importance of perceived control, personal value, and interest for sustained action (Ajzen, 2002, 2005; Ryan and Deci, 2017). Controlled motives were also present. This may reflect the demands of an intensive scholarship program, including examinations, academic expectations, and future study plans. These results should be read as group-level tendencies, since individual learner experiences may differ.

The measurement results generally supported the use of the adapted bilingual scales. Reliability and convergent validity were adequate to strong, and collinearity was low. Discriminant validity was adequate for most construct pairs. However, the overlap between Intrinsic Motivation and Identified Regulation was high. This suggests that learners may have experienced interest in Arabic and personal value for learning Arabic as closely connected reasons. Therefore, results involving these two autonomous motivation variables should be interpreted with caution. The GSCA fit indices also provided mixed evidence. FIT, AFIT, and FITm suggested meaningful explanatory quality, but the estimated SRMR of 0.12 showed that global residual fit was not ideal.

At the same time, the size of several structural associations requires caution. The paths from PBC to Growth Mindsets, from Growth Mindsets to Intrinsic Motivation, and from Growth Mindsets to Identified Regulation were very large for cross-sectional self-report data. These large coefficients may reflect strong conceptual proximity among perceived control, growth-oriented beliefs, and autonomous motivation in this intensive L2 Arabic context. However, they may also partly reflect shared method, the same survey time point, the same response format, and conceptual overlap among the constructs. These coefficients should therefore be interpreted as strong theory-informed associations, not as evidence of strong causal effects.

The structural results showed a clear pattern between PBC and language mindsets. PBC was strongly and positively associated with Growth Mindsets and weakly and negatively associated with Fixed Mindsets. This suggests that learners who felt more capable of managing L2 Arabic learning demands were also more likely to view Arabic ability as improvable. This pattern is consistent with TPB, self-efficacy theory, and mindset theory (Ajzen, 2002; Bandura, 1997; Dweck and Molden, 2017). It also aligns with L2 studies that report positive links between self-efficacy, PBC, and growth language mindsets (Derakhshan and Fathi, 2024; Zarrinabadi et al., 2022; Zhai and Li, 2025). The weaker path to Fixed Mindsets suggests that fixed beliefs may also reflect other influences, such as prior schooling, earlier language learning experiences, and social views about language talent. This supports the idea that growth and fixed beliefs may be partly independent rather than simple opposites (Lou et al., 2022).

The two mindset constructs showed different links with SDT motivation. Fixed Mindsets were positively associated only with External Regulation. Learners who viewed Arabic ability as stable appeared more likely to rely on external demands, such as grades, requirements, or institutional expectations. This finding is consistent with research linking fixed beliefs to evaluation concerns and performance protection (Kırmızı et al., 2023; Lou and Noels, 2016, 2019). However, Fixed Mindsets were not significantly related to Intrinsic Motivation, Identified Regulation, or Introjected Regulation. This suggests that fixed beliefs may not directly reduce autonomous motivation in this context. Some learners may still enjoy Arabic or value it, even when they hold some fixed beliefs about ability.

Growth Mindsets had a broader motivational role. As expected, Growth Mindsets were strongly associated with Intrinsic Motivation and Identified Regulation. Learners who viewed Arabic ability as improvable appeared more likely to enjoy learning Arabic and to see it as personally valuable. This pattern is consistent with SDT, which links competence-related beliefs with more autonomous motivation (Ryan and Deci, 2017, 2020). It also aligns with L2 studies linking growth mindsets with engagement, enjoyment, and internalized learning goals (Jiang et al., 2024; Li et al., 2025; Sadoughi et al., 2023).

However, H9 and H10 were not supported. Growth Mindsets were positively associated with Introjected Regulation and External Regulation, rather than negatively associated with them. This finding is important because it suggests that growth beliefs do not always reduce controlled motivation. In this scholarship-based context, growth-oriented learners may combine autonomous motives with pressure-based motives. They may enjoy Arabic and value it personally, while also feeling responsible to pass levels, maintain progress, meet scholarship expectations, and prepare for future degree study.

This pattern can be understood within SDT. Introjected Regulation refers to internal pressure, such as guilt, shame, fear of failure, or the need to prove oneself. External Regulation refers to outside demands, such as grades, requirements, rewards, or institutional expectations (Ryan and Deci, 2017, 2020; Takahashi and Im, 2020). In the present context, students who believe that Arabic ability can improve may feel that success is possible through effort. This belief may support interest and personal value. At the same time, it may make failure feel more personally accountable. Thus, Growth Mindsets may support adaptive persistence, but they may also increase self-pressure in demanding programs.

This does not mean that Growth Mindsets are negative. Rather, it suggests that their role may depend on context. Lou and Noels (2019) describe language mindsets as part of a wider meaning system linked to learners’ interpretations, emotions, and actions. In lower-pressure settings, Growth Mindsets may mainly support autonomous motivation. In high-stakes scholarship settings, the same beliefs may also make learners more responsive to internal and external demands. The present findings therefore suggest a mixed motivational profile. Growth-oriented learners may combine enjoyment, personal value, self-pressure, and external expectations.

The indirect-path results further showed that PBC was statistically related to motivation mainly through Growth Mindsets. The largest indirect paths linked PBC to Intrinsic Motivation and Identified Regulation through Growth Mindsets. This finding is consistent with the view that mindsets can work as meaning systems related to learners’ interpretations and motivation patterns (Lou and Noels, 2017, 2019; Zarrinabadi et al., 2022). In contrast, indirect paths through Fixed Mindsets were limited and mainly involved External Regulation. This suggests that Growth Mindsets may be a more central link between perceived control and motivation in intensive L2 Arabic learning, while Fixed Mindsets may be more closely tied to externally driven study.

The findings suggest several practical implications. In classrooms, teachers may support PBC and Growth Mindsets by making progress visible and manageable. In beginner courses, Arabic script, sound-symbol links, vocabulary, and basic reading can be divided into short steps. In grammar courses, complex features such as verb patterns, agreement, case marking, and sentence order can be taught through guided tasks with clear models. Feedback should focus on progress, strategy use, and next steps, not only on correctness. For example, teachers can explain errors in pronunciation, word order, or verb forms as part of learning and show one clear way to improve. Short progress records, weekly self-checklists, and learning portfolios may also help students see how effort and strategy use lead to improvement (Bandura, 1997; Dweck and Leggett, 1988; Lou and Noels, 2019).

At the program level, external demands can be connected more clearly to students’ personal and academic goals. Many scholarship students need to pass level examinations and prepare for future Arabic-medium study. These demands may create pressure, but they can also be framed as meaningful preparation. Teachers can show how academic vocabulary, lecture listening, note-taking, and oral presentation skills support later study in fields such as Islamic Studies, Arabic Language Studies, and Computer Sciences. Program leaders can also link placement tests, level outcomes, and examinations to clear learning targets. Short advising sessions may help students review progress and set realistic goals for reading, writing, listening, and speaking. These practices may support learners’ sense of control, encourage growth-oriented beliefs, and help students manage both internal motives and external expectations.

### Limitations and future research

Several limitations should be noted. First, the study used a cross-sectional survey design. This design was appropriate because the study aimed to examine learners’ current beliefs, perceptions, and motivation in a natural educational setting. Questionnaires are widely used in L2 research because they can collect a large amount of information in a short time and in a form that can be analyzed systematically (Dörnyei and Taguchi, 2010). They are also useful for measuring attitudinal constructs, including beliefs, opinions, interests, and values. Brown (2001) also presents survey research as a useful approach in language programs. Thus, the design was well aligned with the study’s focus on PBC, language mindsets, and SDT motivation.

At the same time, the cross-sectional design limits the scope of inference. The findings should be interpreted as theory-informed associations, not as evidence of causal order or change over time. Although the model placed PBC before language mindsets and motivation based on theory, the data were collected at one point in time. Therefore, temporal sequence cannot be confirmed. The indirect paths should also be understood as statistical mediation within the specified model, not causal mediation. Future studies may build on the present findings by using longitudinal, experimental, or mixed-methods designs to examine whether changes in PBC and language mindsets are followed by changes in SDT motivation over time.

Second, the sample included only male students from one public university in southern Saudi Arabia. This reflected the admission structure of the department at the time of data collection, as the program admitted only male students. Therefore, the findings should not be generalized to all L2 Arabic learners, female learners, or all Saudi universities. The results are best understood as evidence from one intensive, scholarship-based L2 Arabic program. Still, the sample included learners from more than 30 national backgrounds and four program levels, which provides useful insight into a diverse international student group. Future research should include female learners, students from several universities, and learners in different Arabic program types.

Third, common method variance cannot be fully ruled out. The study used procedural steps to reduce this risk, including anonymity, voluntary participation, bilingual items, piloting, and screening for unengaged responses. Harman’s single-factor diagnostic also suggested that one factor did not dominate the data. However, all constructs were measured through self-report in one survey session and with the same response format. This issue is especially important because several path coefficients were unusually large. These large coefficients may reflect meaningful associations among perceived control, growth mindsets, and motivation, but they may also reflect common method variance or conceptual closeness among the constructs. Future studies could use additional data sources, such as interviews, classroom observations, learning analytics, achievement records, or longitudinal repeated measures.

Fourth, Intrinsic Motivation and Identified Regulation were highly correlated. Although these constructs are theoretically distinct in SDT and were measured with separate item sets, their empirical separation was limited in this sample. This means that findings involving these two variables should be interpreted with particular caution. In this L2 Arabic scholarship context, learners may have experienced enjoyment, personal value, academic goals, and future study plans as closely connected reasons for learning Arabic. Future studies should examine whether these constructs are better modeled separately or as part of a higher-order autonomous motivation factor in L2 Arabic contexts. An additional limitation is that the adapted bilingual measures were not examined through a separate factor-based CFA. The component-based GSCA results provide measurement evidence within the specified model, but they do not establish how well an equivalent common-factor structure would fit the data. Future studies should test the adapted measures through CFA with an independent L2 Arabic sample. They should also compare separate, combined, and higher-order representations of Intrinsic Motivation and Identified Regulation.

A further limitation concerns model fit. The estimated SRMR was 0.12, which suggests that the specified model did not show ideal global residual fit. Although GSCA fit evaluation differs from covariance-based SEM, this result indicates that some covariance patterns may not have been fully represented by the model. Therefore, the model fit evidence should be interpreted as mixed rather than strong. Future studies should test theoretically justified alternative models, examine residual patterns, and assess whether the same structure is stable in larger and more diverse L2 Arabic samples.

Finally, the unexpected positive links between Growth Mindsets and controlled motivation require further study. The present findings suggest that growth-oriented learners in scholarship-based programs may combine autonomous motives with internal and external pressure. Future longitudinal and qualitative research should examine whether this profile supports adaptive persistence, increases pressure-based striving, or produces both outcomes over time.

## Conclusion

This study extends language learner psychology research to an underrepresented L2 Arabic context by examining PBC, language mindsets, and SDT motivation in one hierarchical model. The findings suggest that PBC was strongly associated with Growth Mindsets and showed indirect associations with both autonomous and controlled motivation. Fixed Mindsets were mainly linked to External Regulation and showed a more limited indirect role. The results also suggest that context matters. In high-stakes scholarship programs, growth-oriented learners may sustain effort through interest and personal value, but also through internal pressure and external expectations.

The findings should be interpreted with caution because the study was cross-sectional, relied on self-report data, and included male learners from one university. Intrinsic Motivation and Identified Regulation also showed high overlap. Even with these limits, the study offers useful evidence for intensive L2 Arabic programs. Supporting learners’ sense of control, making progress visible, and encouraging growth-oriented beliefs may help sustain motivation and progress among international L2 Arabic learners.

## Data Availability

The raw data supporting the conclusions of this article will be made available by the authors, without undue reservation.
